# Supplementary material for: The genome sequence of the Buff Footman, Eilema depressum (Esper, 1787)
Source: Wellcome Open Res. 2023 Aug 11;8:342. [Version 1] doi: 10.12688/wellcomeopenres.19808.1 (PMC11322702; doi:10.12688/wellcomeopenres.19808.1)
Supplement: Peer review history

# Reviewer report: Reviewer response for version 1

**DOI:** 10.21956/wellcomeopenres.21940.r93173

**Published:** 2024-08-28

**Authors:** Lapo Ragionieri

**Affiliations:** 1 University of Cologne, Cologne, Germany

The data note titled "The genome sequence of the Buff Footman, *Eilema depressum* (Esper, 1787)" by Douglas Boyes, Chelsea Skojec, and Akito Y. Kawahara describes the sequencing and genome assembly of *Eilema depressum*. The authors utilized long-read sequencing technology (PacBio) to produce an initial assembly, which was subsequently scaffolded using Hi-C, resulting in a genome assembly at the chromosome level, with nearly all scaffolds assigned to chromosomes. The genome assembly has a total length of 622.0 Mb across 30 chromosomes. There are some discrepancies between the text and the tables that should be corrected. The genome annotation includes 20,038 protein-coding genes.

 The presentation of the results is well done, but the authors did not report any information about the masking of the genome.

**Below are some specific comments and suggestions:**

1. **Keywords**: The keywords should be distinct from the title to be effective.
2. **Species Identification**: The authors should cite the literature used for species identification.
3. **Mitochondrial Genome**: I strongly recommend submitting the mitochondrial genome separately with an independent accession number. Moreover, while the authors described the methods, they did not provide information about the mitochondrial genome annotation (coding genes, rRNAs, and tRNAs). It is expected that the authors submit the annotations obtained by MITOS separately.
4. **Annotation**: The authors should identify and quantify the transposable elements in the genome.
5. **Command Lines**: It is common practice to include the command lines used with all software. Providing this information would be very useful for readers.

Are sufficient details of methods and materials provided to allow replication by others?

Yes

Is the rationale for creating the dataset(s) clearly described?

Yes

Are the datasets clearly presented in a useable and accessible format?

Yes

Are the protocols appropriate and is the work technically sound?

Yes

Reviewer Expertise:

genomic transcriptomics and proteomics

I confirm that I have read this submission and believe that I have an appropriate level of expertise to confirm that it is of an acceptable scientific standard.

# Reviewer report: Reviewer response for version 1

**DOI:** 10.21956/wellcomeopenres.21940.r93172

**Published:** 2024-08-23

**Authors:** Yang Mei

**Affiliations:** 1 State Key Laboratory of Rice Biology & Ministry of Agricultural and Rural Affairs Key Laboratory of Molecular Biology of Crop Pathogens and Insect Pests, Institute of Insect Sciences, Zhejiang University, Hangzhou, Zhejiang, China

This manuscript presents the genome assembly of the Buff Footman moth, *Eilema depressum*. The authors employ a rigorous approach to sample collection, nucleic acid extraction, and sequencing, incorporating advanced techniques such as HiFi and Hi-C. Using robust assembly methods, they have successfully generated a high-quality genome, achieving an impressive BUSCO completeness score of 98.8% and a scaffold N50 of 22.1 Mb.

 The manuscript is well-structured, with data and methodologies that are both appropriate and reliable. In my opinion, this work is well-suited for indexing in Wellcome Open Research.

 That said, there are a few minor concerns that merit clarification: specifically, why was a male (ZZ karyotype) sample chosen for sequencing instead of a female (ZW karyotype)?

Are sufficient details of methods and materials provided to allow replication by others?

Yes

Is the rationale for creating the dataset(s) clearly described?

Yes

Are the datasets clearly presented in a useable and accessible format?

Yes

Are the protocols appropriate and is the work technically sound?

Yes

Reviewer Expertise:

Insect genomics

I confirm that I have read this submission and believe that I have an appropriate level of expertise to confirm that it is of an acceptable scientific standard.

# Reviewer report: Reviewer response for version 1

**DOI:** 10.21956/wellcomeopenres.21940.r93175

**Published:** 2024-08-13

**Authors:** Komal Kumar Bollepogu Raja

**Affiliations:** 1 Department of Pathology & Immunology, Baylor College of Medicine, Houston, TX, USA

The article by Boyes et al *.,* presents genome sequence for *Eilema depressum*. The authors provide comprehensive and detailed data on the assembly, annotation, methods and quality of the genome for this moth species. The final assembly has a high Quality Value (QV) of 60.7, with 100% k-mer completeness and a BUSCO completeness score of 98.8%. These metrics indicate a well-assembled genome with a low level of fragmentation. Furthermore, the scaffolds are well-documented with a total length of 622.0 Mb and 30 chromosome-scale scaffolds. This detailed work supports further research into community dynamics and secondary compound sequestration in lichen-feeding moths. Overall, the data is of high quality and the manuscript is well written.

**Minor comment:**

 The authors mention that contigs of second haplotype were deposited separately. It would benefit if the authors add text noting differences between the haplotypes and if they affect analyses.

Are sufficient details of methods and materials provided to allow replication by others?

Yes

Is the rationale for creating the dataset(s) clearly described?

Yes

Are the datasets clearly presented in a useable and accessible format?

Yes

Are the protocols appropriate and is the work technically sound?

Yes

Reviewer Expertise:

Genomics, Bioinformatics, Computational biology, single cell omics

I confirm that I have read this submission and believe that I have an appropriate level of expertise to confirm that it is of an acceptable scientific standard.

# Reviewer report: Reviewer response for version 1

**DOI:** 10.21956/wellcomeopenres.21940.r88932

**Published:** 2024-08-06

**Authors:** Sivasankaran Kuppusamy

**Affiliations:** 1 Entomology Research Institute, Loyola College, Chennai, India

Authors have sequenced and assembled the whole genome of Buff Footman moth *Eilema depressum* (Esper, 1787). A total length of 622 mb sequence was assembled. A total of 30 chromosomes were confirmed through the assembly.  Totally 20, 038 protein coding genes and 20, 236 gene transcripts were observed through the assembly and annotation.

**Minor comments on the manuscripts:**

- Authors have given the genus name of the species *Eilema depressum* full form throughout the article. And given a short form somewhere. First time the genus name can be given in full form then subsequently can be given in short form like *E. depressum*.
- In the background the first sentence can be modified as “The Buff Footman moth, *Eilema depressum* (Esper, 1787) is a drab-coloured moth belonging to the family *Erebidae*”.
- In the genome sequence report (subheading), the first line can be modified as “The genome was sequenced from a male *Eilema depressum* specimen (Figure 1)”. The collection details need not be given here. Because the collection details were given in the methods.  
- The table 3 legend was given inside text. The legend can be changed as a text “A list of relevant software tool versions and sources are given in the Table 3”.
- Above all, the manuscript was well prepared, and I confirm that the manuscript meets the necessary scientific standard and is suitable for indexing.

Are sufficient details of methods and materials provided to allow replication by others?

Yes

Is the rationale for creating the dataset(s) clearly described?

Yes

Are the datasets clearly presented in a useable and accessible format?

Partly

Are the protocols appropriate and is the work technically sound?

Yes

Reviewer Expertise:

phylogenetic analysis of moths using mitogenome

I confirm that I have read this submission and believe that I have an appropriate level of expertise to confirm that it is of an acceptable scientific standard.
